# Comparative genomic analysis revealed great plasticity and environmental adaptation of the genomes of *Enterococcus faecium*

**DOI:** 10.1186/s12864-019-5975-8

**Published:** 2019-07-22

**Authors:** Zhi Zhong, Lai-Yu Kwok, Qiangchuan Hou, Yaru Sun, Weicheng Li, Heping Zhang, Zhihong Sun

**Affiliations:** 1 Key Laboratory of Dairy Biotechnology and Engineering, Ministry of Education, Inner Mongolia Agricultural University, Hohhot, China; 2 Key Laboratory of Dairy Products Processing, Ministry of Agriculture and Rural affairs, Inner Mongolia Agricultural University, Hohhot, China; 3 Inner Mongolia Key Laboratory of Dairy Biotechnology and Engineering, Hohhot, China

**Keywords:** *Enterococcus faecium*, Genomes, Environmental adaptation, Evolution, Antibiotic resistance genes

## Abstract

**Background:**

As an important nosocomial pathogen, *Enterococcus faecium* has received increasing attention in recent years. However, a large number of studies have focused on the hospital-associated isolates and ignored isolates originated from the natural environments.

**Results:**

In this study, comparative genomic analysis was conducted on 161 isolates originated from human, animal, and naturally fermented dairy products. The results showed that the environment played an important role in shaping the genomes of *Enterococcus faecium*. The isolates from human had the largest average genome size, while the isolates from dairy products had the smallest average genome size and fewest antibiotic resistance genes. A phylogenetic tree was reconstructed based on the genomes of these isolates, which revealed new insights into the phylogenetic relationships among the dairy isolates and those from hospitals, communities, and animals. Furthermore, 202 environment-specific genes were identified, including 136 dairy-specific, 31 human blood-specific, and 35 human gastrointestinal-specific genes. Interestingly, five dairy-specific genes (namely *lacF*, *lacA*/*B*, *lacD*, *lacG*, and *lacC*) that constituted an integrated lactose metabolism pathway existed in almost all dairy isolates. The pathway conservation demonstrated an active role of the environment in shaping the genomes of *Enterococcus faecium*.

**Conclusions:**

This study shows that the *Enterococcus faecium* species has great genomic plasticity and high versatility to occupy broad ecological roles, dwelling as non-harmful dairy and animal gut commensals as well as significant nosocomial pathogens that disseminate antibiotic resistance genes.

**Electronic supplementary material:**

The online version of this article (10.1186/s12864-019-5975-8) contains supplementary material, which is available to authorized users.

## Background

*Enterococci* are common gastrointestinal (GI) commensal bacteria in humans and other animals [1]. However, over the last 30 years, nosocomial infections due to enterococci have been increasing continuously and have become a top leading cause of hospital-acquired infections of the GI tract, bloodstream, and urinary tract (UT) [2–5]. Two *Enterococcus* (*E*.) species, *E. faecalis* and *E. faecium*, are of major clinical concerns. In the late 1970s and 1980s, hospital-acquired enterococcal infections were mainly caused by *E. faecalis* due to their high intrinsic virulence [2]. However, the prevalence of nosocomial infections caused by *E. faecium* is rising since the early 1990s in the hospitals in the United States, and it has even partially replaced *E. faecalis* to become a main culprit of nosocomial infections around the world in recent years [2, 6]. The major reason for *E. faecium* to become a top nosocomial pathogen is its multidrug resistance. Many of the modern nosocomial *E. faecium* isolates are resistant to ampicillin, aminoglycosides, quinupristin–dalfopristin, linezolid, and vancomycin [2]. Vancomycin is the last line of defense against a wide range of multi-resistant Gram-positive pathogens [7]. In recent years, the number of infections associated with vancomycin-resistant enterococci has increased rapidly in many countries. For example, the percentage of vancomycin-resistant *E. faecium* rose from 0% before the mid 1980s to more than 80% by 2007 in the United States [8]. To decipher how *E. faecium* has become a globally disseminated nosocomial pathogen, several whole-genome sequencing-based studies have been performed [9–11]. Phylogenetic analysis based on whole-genome sequencing has shown that the *E. faecium* population is formed by two clades: clade A contains mainly clinical or hospital-associated isolates, while clade B contains primarily commensal/community-associated isolates [12, 13]. Further work has shown that a second split occurred within clade A approximately 75 years ago, resulting in two subclades, subclade A1 and subclade A2. Subclade A1 contains mainly the clinical isolates, including the successful hospital-adapted sequence types of the clonal complex 17 (CC17) genogroup defined by multilocus sequence typing, while subclade A2 mostly consisted of the animal-associated isolates [9]. However, a recent comparative genomic study of health-care associated *E. faecium* from UK and Ireland did not support the subdivision of clade A into the two subclades [11]. Most published studies have so far focused on isolates from clinical settings and animal origins. In fact, *E. faecium* is ubiquitous in nature and has been isolated from a wide range of environments [14]. Thus, to gain deeper insights into the evolutionary origin of *E. faecium*, a large sampling from multiple-habitats is required to better represent the natural distribution of this microbial group.

Apart from human/animal GI tracts and clinical samples, fermented dairy foods are indeed natural habitats for *E. faecium* [15, 16]. Isolates of *E. faecium* originated from fermented dairy products do not only act as bystanders but also participate in the biochemical reactions that occur during the ripening process [17]. Some *E. faecium* isolates are known to confer beneficial effects to the host and are even marketed as commercial probiotics (e.g. *E. faecium* isolate T110). A recent comparative genome analysis has revealed genetic features of potential probiotic *E. faecium* isolates [18]. Providing the probiotic, non-pathogenic non-probiotic, and pathogenic nature among the *E. faecium* isolates, it is therefore interesting to investigate if there are links between their isolation environment, physiological features, and phylogeny.

Another interesting question is if the dairy isolates are a product of faecal contamination or environmental habitat, since the selective pressure for *E. faecium* to survive in dairy products is enormously different from the complex niches within human and animal hosts. The dairy has relative simple carbon and nitrogen source, convergent evolution is likely to be dominant when the bacteria adapt to the dairy environment. Thus, comparative study integrating environmental isolates such as dairy isolates as reference could provide insights to understand not only of the hospital-acquired infection and multidrug resistance of *E. faecium* but also their mechanisms of environmental adaptation.

To gain deeper insights into the genetic relationships of the dairy isolates and the isolates from human and animals, this study performed a comparative genomic analysis on 161 *E. faecium*, of which 54 dairy isolates were isolated and sequenced by our laboratory while the other 107 genome sequences were retrieved from a public genome database.

## Results

### General characteristics of the genomes of *E. faecium*

**Fig. 1:**
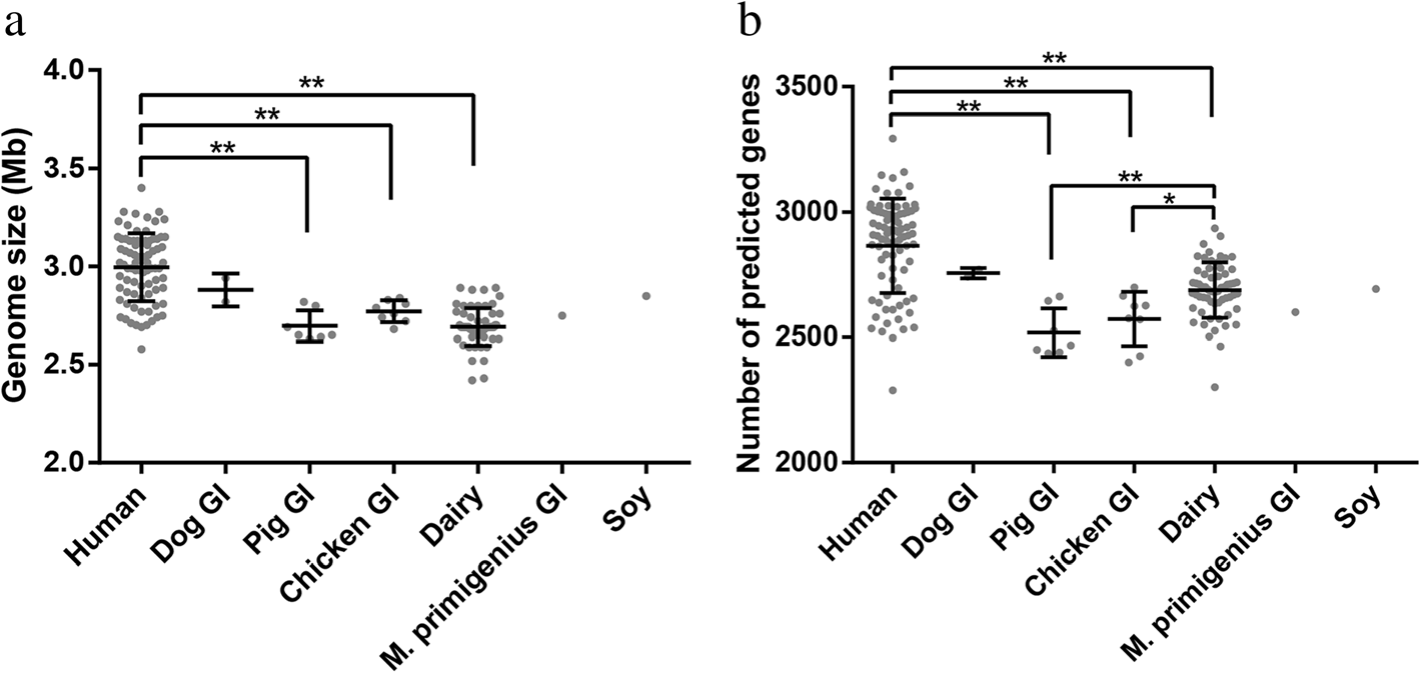
Genome size (**a**) and number of predicted genes (**b**) of *Enterococcus faecium* isolates from different environments. The asterisk (*) and double asterisks (**) represent *p* < 0.05 and < 0.01 from the one-way ANOVA test, respectively

In this study, comparative genomic analysis was conducted on 161 isolates, including 33 isolates from human GI, 39 isolates from human blood, nine isolates from human UT, eight isolates from chicken GI, two isolates from dog GI, seven isolates from pig GI, one isolate from *Mammuthus* (*M*.) *primigenius* GI, 61 isolates from dairy products, and one isolate from soy, respectively. The *E. faecium* genomes had a low overall G + C content ranging from 37.4 to 38.6% (Additional file 1). The average genome size was 2.85 ± 0.20 Mb, with 2,765 ± 187 predicted genes (Additional file 1). Furthermore, apart from the two dog GI-originated isolates, significant differences existed in the genome size and/or the number of predicted genes between isolates from different environments (Fig. 1). Generally, the human-originated isolates had the largest mean genome size and the maximum mean number of predicted genes, which were significantly higher than the isolates of pig GI (*P* < 0.01), chicken GI (P < 0.01), and dairy (P < 0.01). Meanwhile, it is interesting to note that although no significant difference was observed in the genome size among these three groups, significantly more predicted genes were identified in the dairy isolates compared with those originated from pig GI (*P* = 0.006) and chicken GI (*P* = 0.048). Further analysis showed that the dairy isolates had more short genes (100–200 bp) than pig and chicken isolates (Additional file 2). On average, the dairy isolates had 150 fewer predicted genes compared with those of human origin. The missing genes represented mainly the COG functional categories of replication, recombination and repair [L] and carbohydrate transport and metabolism [G] (Additional file 3). Since the sample size of both the *M. primigenious* GI and soy groups was too small (*n* = 1 in each case), no statistical analysis could be performed.

**Fig. 2:**
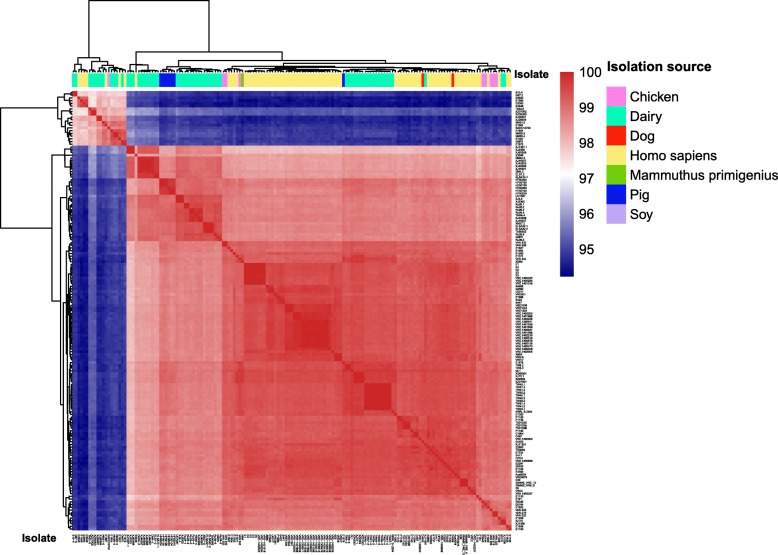
The heatmap of the average nucleotide identity of 161 *Enterococcus faecium* isolates

To evaluate the genetic difference between strains, we generated an average nucleotide identity (ANI) plot (Fig. 2). The ANI values across all strains ranged from 94.23 to 99.99, with the lowest ANI value between the isolates E3548 (human blood) and TW5–3 (dairy). The two dairy isolates, had the highest ANI (99.99). The ANI values of the dairy and human isolates ranged from 94.53 to 99.99 and 94.27 to 99.99, respectively. The genetic closeness between strains could be classed into two groups according to the ANI value (20 isolates with a lower overall ANI value, while the other 141 isolates had a higher overall ANI), although no obvious grouping pattern was noted based on the bacterial isolation source (Fig. 2).

### The pan- and core-genome of *E. faecium*

**Fig. 3:**
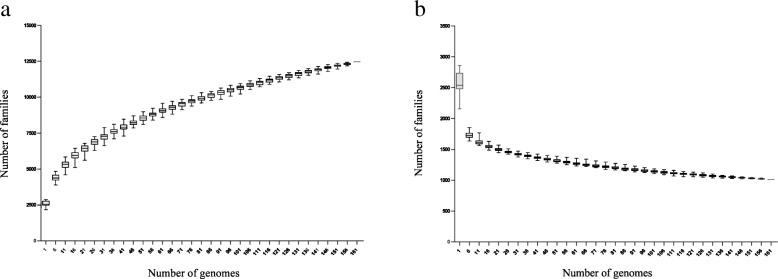
Pan-genomes (**a**) and core-genomes (**b**) of the species *Enterococcus faecium.* The gene accumulation curves describe the number of genes obtained by adding a new genome to a previous set. The procedure was repeated 1,000 times by randomly modifying the order of integration of genomes

The pan-genome of the 161 *E. faecium* isolates comprised 12,457 gene families, and the pan-genome size increased with the number of genomes (Fig. 3a). In contrast, the core-genome size gradually decreased with the addition of deciphered genomes. When the number of genomes reached 130–140, the core-genome size remained stable (Fig. 3b). The core gene set contained 1013 genes, corresponding to 36.6% of the average number of predicted genes per genome (2765 genes). In other words, almost two-thirds of the predicted genes in each genome were accessory.

The results of the functional analysis of the pan- and core-genome are shown in Table 1. Around one-third (33.4%) of the core genes were distributed to the COG functional categories of translation, ribosomal structure and biogenesis [J]; amino acid transport and metabolism [E]; transcription [K]; and carbohydrate transport and metabolism [G]. The pan genes mainly fell into categories representing the COG categories: carbohydrate transport and metabolism [G]; cell wall/membrane/envelope biogenesis [M]; replication, recombination and repair [L]; and transcription [K]. The COG category genes of carbohydrate transport and metabolism [G] were enriched in both the pan- and core-genomes. However, it was the most abundant COG category class (12.74%) detected only within the pan-genome but not the core-genome (7.36%), and it resulted in a large pan-genome expansion of 9.72-fold, suggesting strong selective pressure in these genes and niche selection.

**Table 1:** Functional categories of representative core and pan genes of *Enterococcus faecium* genomes

COG Functional category	No. of core genes	No. of pan genes	Proportion of core genes among pan genes (%)
[J] Translation, ribosomal structure and biogenesis	113	435	25.98
[A] RNA processing and modification	0	0	0
[K] Transcription	79	506	15.61
[L] Replication, recombination and repair	55	515	10.68
[B] Chromatin structure and dynamics	0	0	0
[D] Cell cycle control, cell division, chromosome partitioning	15	61	24.59
[Y] Nuclear structure	0	0	0
[V] Defense mechanisms	16	200	8.00
[T] Signal transduction mechanisms	31	204	15.20
[M] Cell wall/membrane/envelope biogenesis	49	539	9.09
[N] Cell motility	2	24	8.33
[Z] Cytoskeleton	0	0	0
[W] Extracellular structures	0	0	0
[U] Intracellular trafficking, secretion, and vesicular transport	12	229	5.24
[O] Post-translational modification, protein turnover, and chaperones	34	102	33.33
[C] Energy production and conversion	36	289	12.46
[G] Carbohydrate transport and metabolism	75	729	10.29
[E] Amino acid transport and metabolism	81	263	30.80
[F] Nucleotide transport and metabolism	52	91	57.14
[H] Coenzyme transport and metabolism	29	84	34.52
[I] Lipid transport and metabolism	31	85	36.47
[P] Inorganic ion transport and metabolism	63	207	30.43
[Q] Secondary metabolites biosynthesis, transport, and catabolism	9	55	16.36
[R] General function prediction only	136	545	24.95
[S] Function unknown	101	559	18.07

### Phylogenetic reconstruction of *E. faecium*

**Fig. 4:**
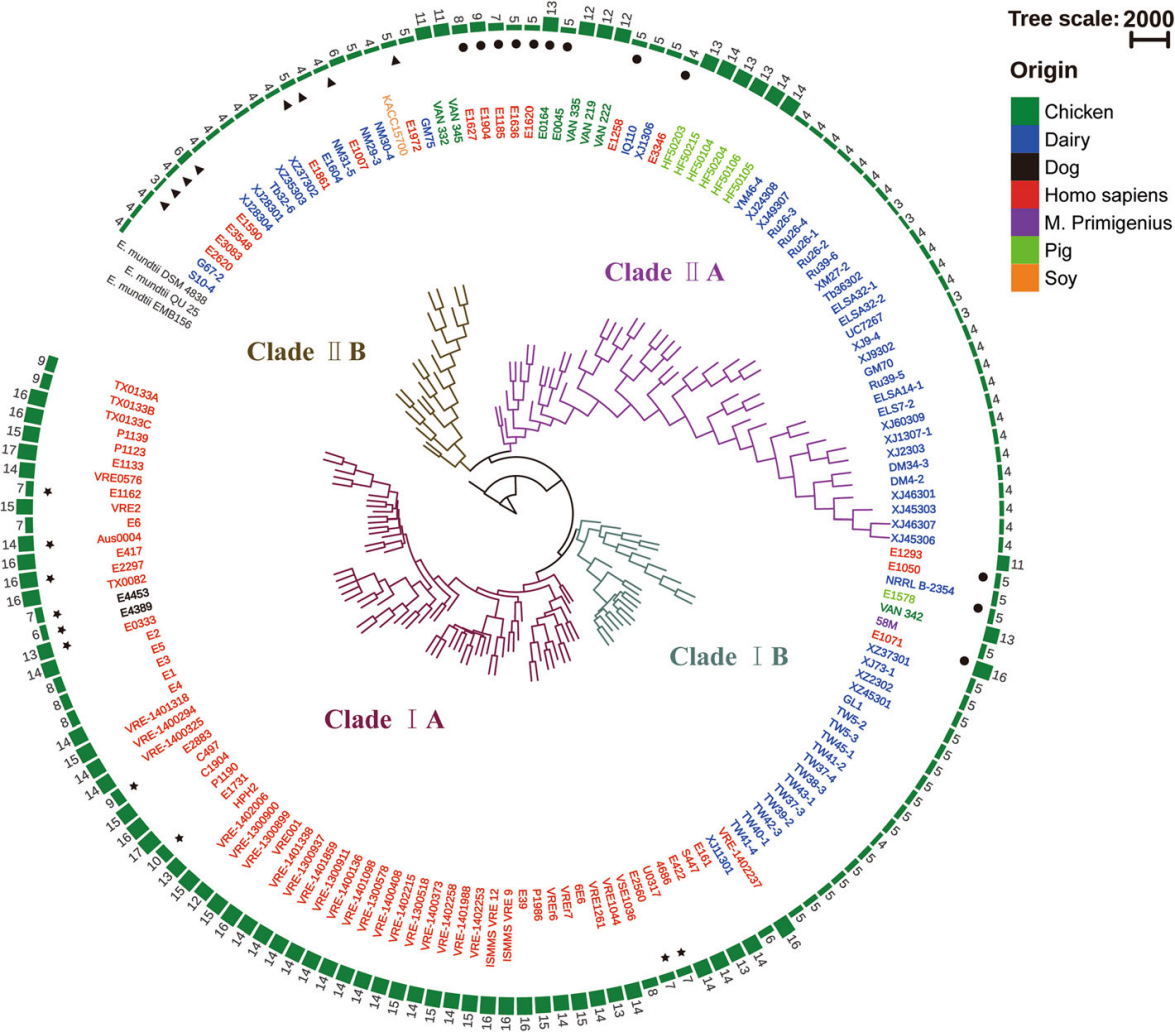
Phylogenetic tree constructed based on the core genes of *Enterococcus* (*E*.) *faecium* isolates. The phylogenetic tree was constructed using the DNA sequences of 871 core genes of 161 isolates of *E. faecium* and 3 isolates of *E. mundti*. *Enterococcus mundtii* is the closest phylogenetic relative of *E. faecium* and was thus included as outgroup. The color of the isolate name represents the origin of the isolate. Isolates labeled with star, dot, and triangle in the out circle were clustered in clade A1 (hospital-associated), clade A2 (animal-associated), and clade B (commensal-associated), respectively, in Lebreton et al. [9]. The number of antibiotic resistance genes was labeled in the out circle

A phylogenetic tree was constructed to investigate the evolutionary relationship among the studied isolates (Fig. 4, Additional file 4). Here three isolates of *E. mundtii*, the closest phylogenetic relatives of *E. faecium*, were included as outgroups. The topology of the phylogenetic tree revealed two clades, which were defined as clades I and II in this study (Fig. 4). Furthermore, two subclades could be identified within clade I, defined as subclades IA and IB. Subclade IA was larger and comprised mostly the human isolates (64 of the 67 isolates), as well as the two isolates from dog GI and one isolate from a dairy product of Xinjiang, China. Subclade IB contained 24 isolates. The majority of isolates from this subclade were isolated from dairy products, including all the dairy isolates from Tyva, Russia. In addition, three isolates from human, one isolate from pig, one isolate from chicken, and the isolate from *M*. *primigenius* (isolate 58 M) also belonged to this subclade. Similarly, two subclades could be identified within clade II, defined as subclade IIA and IIB. Subclade IIA contained 50 isolates, including 30 isolates from dairy, seven isolates from human, and almost all animal-originated isolates (seven isolates from chicken and six isolates from pig). Interestingly, most of the human and animal isolates were close to the base of this subclade, while the dairy isolates were mainly in the crown. Subclade IIB was hybrid containing human and dairy isolates. The isolate from soy, KACC15700, was also allocated to this subclade.

To allow comparison between the current results and previously published works in the field, this study included genomes of 30 isolates reported in Lebreton et al. [9]. In Lebreton et al., these isolates were divided into two separate clades (i.e. clade A and clade B), and a relatively recent split occurred within clade A to form clade A1 (containing mainly hospital-associated isolates) and clade A2 (containing mainly animal-associated isolates). However, 12 animal-associated isolates that were assigned to clade A2 in Lebreton et al. (indicated with dots in Fig. 4) were found to be dispersed in clade I (three isolates) and clade II (nine isolates) in the currently constructed phylogenetic tree, respectively, suggesting a longer genetic distance among these isolates than previously reported.

### Antibiotic resistance genes

Potential antibiotic resistance genes were detected by blasting the 161 *E. faecium* genomes against the CARD database (Additional file 5). A total of 34 antibiotic resistance genes were found within the investigated *E. faecium* genomes (i.e. average of 8.9 antibiotic resistance genes per genome). The number of antibiotic resistance genes varied greatly between isolates. The isolates P1190 (human blood) and E1133 (human GI) contained the largest number of antibiotic resistance genes (17 antibiotic resistance genes in each case), while four dairy isolates (Ru39–6, XJ9302, XJ9–4, and XM27–2) and one isolate from human blood (E3083) had the least antibiotic resistance genes (only three). The dairy isolates had the fewest antibiotic resistance genes, i.e. only 4.4 antibiotic resistance genes per genome on average, which was significantly lower than that of the human, chicken, and pig isolates (*P* < 0.01). Interestingly, the isolate 58 M from *M. primigenius* GI had only five antibiotic resistance genes, which was apparently fewer than other GI isolates (i.e. an average of 12.1 genes per genome).

Although the distribution of antibiotic resistance genes varied greatly between isolates, five antibiotic resistance genes were commonly present in the investigated isolates. The outer membrane factor of the *AdeABC* multidrug efflux complex, *AdeC*, was present in all investigated isolates. The aminoglycoside acetyltransferase, AAC (6′)-Ii, was found across 160 isolates; this gene contributes to the intrinsic resistance to several aminoglycosides [19]. It is an important microbial resistance determinant of *E. faecium* [20]. The dihydrofolate reductase, *DfrE*, was detected in 158 isolates. The ABC-efflux pump, MsrC, occurred in 152 isolates. This gene confers resistance to erythromycin and other macrolide and streptogramin B antibiotics. The major facilitator superfamily (MFS) transporter permease, EfmA, was found in 108 strains.

**Fig. 5:**
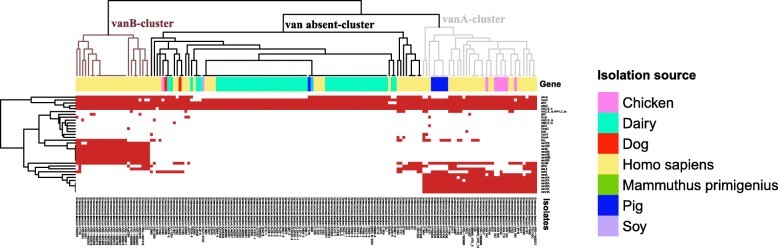
Cluster analysis based on the profile of antibiotic resistance genes Red dots represent genes present in the individual isolates. Three clusters could be identified based on the distribution of vancomycin resistance genes: *vanA*-cluster, *vanB*-cluster, and van absent-cluster

A cluster analysis based on the distribution of the vancomycin resistance genes was performed to investigate if the profile of antibiotic resistance genes was associated with the isolation source (Fig. 5). Three distinct clusters (namely *vanA*-cluster, *vanB*-cluster, and van absent-cluster) could be identified based on the distribution of the vancomycin resistance genes. *VanA*-cluster contained 40 isolates, including 15 isolates from human blood, six isolates from human GI, six isolates from human UT, seven isolates from chicken GI, and six isolates from pig GI. In addition to the five common antibiotic resistance genes, members of the *vanA*-cluster were characterized by possessing a *vanA*-type gene cluster, which was composed of six vancomycin-resistant genes: *vanZA*, *vanYA*, *vanXA*, *vanHA*, *vanSA*, and *vanRA*. Twenty-six isolates belonged to *VanB*-cluster, including six isolates from human blood, 19 isolates from human GI, and one isolate from human UT. Apart from the five common antibiotic resistance genes, members of this cluster were characterized by a *vanB*-type gene cluster that contained six vancomycin-resistant genes: *vanRB*, *vanSB*, *vanYB*, *vanWB*, *vanHB*, and *vanXB*. Ninety-five isolates were assigned to van absent-cluster, including all isolates from dairy, *M. primigenius* GI, and soy. Members of this cluster had the fewest antibiotic resistance genes and were absent of vancomycin resistance genes. Moreover, most members possessed only the five common antibiotic resistance genes.

### Virulence factors

The virulence factors present in the 161 *E. faecium* genomes were identified by a blast search against the VFDB database. A total of 10 putative virulence factors (3.1 per genome) were detected within the 161 *E. faecium* genomes (Additional file 6). The isolates VRE-1400325 (human GI) had the highest number of virulence factors (6 per genome). On average, the number of virulence genes of human isolates (3.5 per genome) was significantly higher than those from dairy (2.7 per genome, *P* < 0.01), pig GI (2.4 per genome, P < 0.01), and chicken GI (2.5 per genome, P < 0.01). The isolate 58 M from *M. primigenius* GI had three virulence genes, while the two isolates from dog GI had 3.5 virulence genes per genome.

In summary, the virulence factors of *E. faecium* were mainly adhesion proteins that mediate adherence to host tissues, including *efaA* (an endocarditis specific antigen, present in 160 isolates), *acm* (a collagen adhesin precursor, present in 93 isolates), *ecbA* (a collagen binding MSCRAMM, present in 42 isolates), *ebp pili* (endocarditis- and biofilm-associated pili, present in 14 isolates), and *esp* (an enterococcal surface protein, present in 9 isolates). In addition to adhesion proteins, *bopD* (a *LacI* family transcriptional regulator that associates with biofilm formation) was another common virulence factor, which was present in 123 isolates.

### Environment-specific genes

**Fig. 6:**
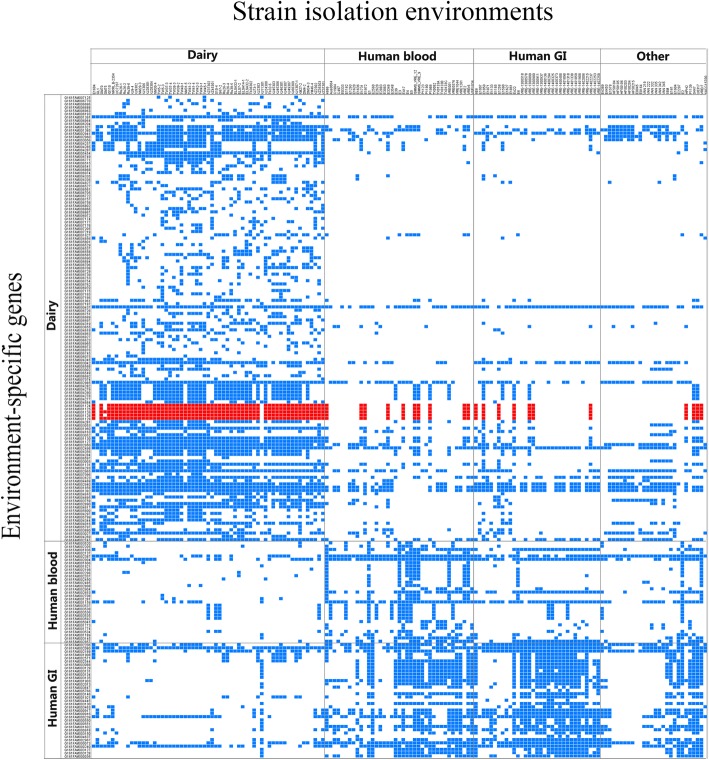
Heatmap of environment-specific genes Blue dots represent genes present in the individual isolates. Genes associated with lactose metabolism are highlighted in red

A genome-wide association study was carried out to identify environment-specific genes among the *E. faecium* isolates. This analysis only included isolates originated from human blood, human GI, and dairy. Isolates from human UT, chicken GI, dog GI, pig GI, *M. primigenius* GI, and soy were excluded from this analysis due to the small sample size. In total, 363 environment-specific genes were initially identified with Scoary. Since Scoary employs the pairwise comparisons algorithm to identify the maximum number of phylogenetically non-intersecting pairs of isolates that contrast in the state of both genotype and phenotype, genes present in a single branch of the phylogenetic tree were removed manually to avoid the problem of lineage-specific interdependencies. Two-hundred and two environment-specific genes remained after the filtration step, including 136 dairy-specific, 31 human blood-specific, and 35 human GI-specific genes (Fig. 6, Additional file 7). Functional analysis was performed on the environment-specific genes using the NCBI nr, COG, and KEGG databases. The identified environment-specific genes were mainly associated with carbohydrate transport and metabolism [G]; replication, recombination and repair [L]; cell wall/membrane/envelope biogenesis [M]; energy production and conversion [C]; coenzyme transport and metabolism [H]; and inorganic ion transport and metabolism [P]. Ninety of the environment-specific genes encoded hypothetical proteins.

**Fig. 7:**
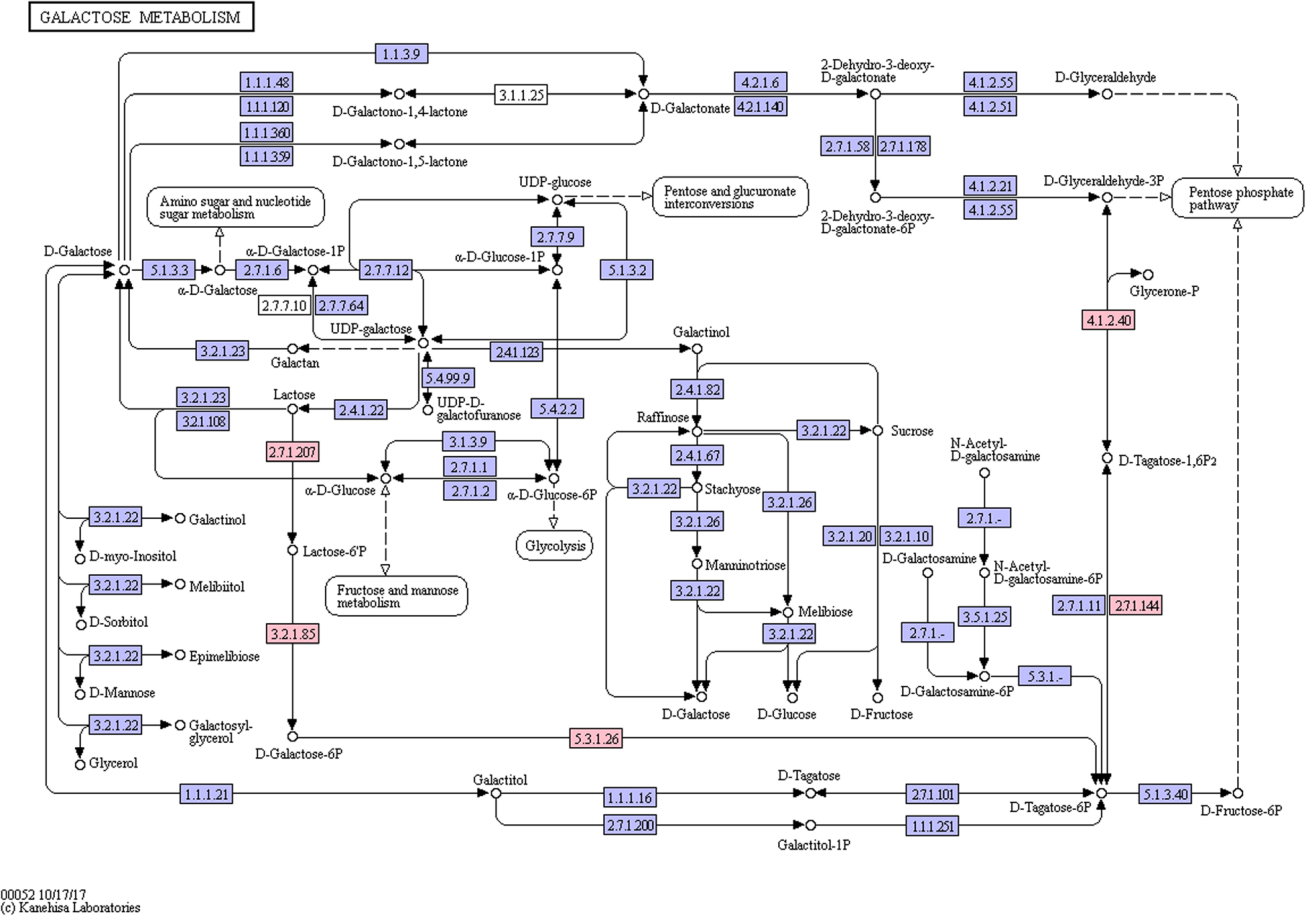
An integrated lactose metabolism pathway constituted by five dairy-specific genes, namely *lacF* [EC:2.7.1.207], *lacG* [EC:3.2.1.85], *lacA*/*B* [EC:5.3.1.26], *lacC* [EC:2.7.1.144], and *lacD* [EC:4.1.2.40] (highlighted in pink)

Sixteen of the dairy-specific genes were involved in carbohydrate transport and metabolism. Among them, the most prevalent ones were *lacF* [EC:2.7.1.207], *lacA*/*B* [EC:5.3.1.26], and *lacD* [EC:4.1.2.40] (present in 59 of the 61 dairy isolates), as well as *lacG* [EC:3.2.1.85] and *lacC* [EC:2.7.1.144] (present in 58 of the 61 dairy isolates). It is noteworthy that these five genes constitute an integrated lactose metabolism pathway (Fig. 7), which facilitates intracellular transport and utilization of extracellular lactose. In addition, five dairy-specific genes function in energy production and conversion, namely citrate lyase ligase, citrate lyase subunit alpha, citrate lyase subunit gamma, citrate sodium symporter, and malic enzyme family protein. Three genes were involved in inorganic ion transport and metabolism, encoding a cadmium-exporting ATPase, a copper chaperone, and a magnesium transporter.

The human blood-specific genes included four carbohydrate transport and metabolism-associated genes that participate in the fructose and tagatose metabolism, three cell wall/membrane/envelope biogenesis-associated genes (i.e. glycosyl transferase, glycosyl transferase family 1, and tagatose-6-phosphate ketose isomerase), two bacteriocin-associated genes, and one transposase-encoding gene.

The human GI-specific genes included three carbohydrate transport and metabolism genes (i.e. N-acylglucosamine-6-phosphate 2-epimerase, PTS glucose transporter subunit IIBC, and spermidine/putrescine ABC transporter permease), three transposases that function in DNA recombination, three transcriptional regulators, two coenzyme transport and metabolism-associated genes (i.e. 3-methyl-2-oxobutanoate hydroxymethyltransferase and pantoate-beta-alanine ligase).

## Discussion

This study performed comparative genomic analysis on 161 *Enterococcus faecium* isolates, and our data showed that the dairy isolates were genetically distinct from those of human origin, supporting the presence of a high diversity and adaptability within the *E. faecium* species.

*Enterococcus faecium* has drawn wide attention in recent years because of its increasing clinical significance in causing nosocomial infections [2]. Moreover, drastic increase in the prevalence of vancomycin-resistant *E. faecium* has been observed in many countries. On the other hand, certain *Enterococcus faecium* strains are used in food and feed production because of their potential beneficial effects [18], although this species is not generally regarded as safe (GRAS) [21]. By taking advantage of the advent in genome sequence technology and comparative genomic analysis, our work deciphered the intraspecific genetic relationships among *E. faecium* isolates, particularly identified structural and functional genomic features that help distinguish between clinical and non-clinical members of this species.

Firstly, some general features of the *E. faecium* genomes were analyzed. The dairy isolates had a significantly smaller genome size and number of predicted genes compared with the human isolates. Although the isolates from dairy, pig, and chicken had no significant difference in genome size, the average number of predicted genes was significantly higher for the dairy isolates mainly due to significantly more short genes (100–200 bp) present in the dairy isolates compared with the pig and chicken isolates. These results suggest apparent distinction between the dairy isolates from those of other origins.

Secondly, phylogenetic analysis was performed to explore the evolutionary relationship among isolates originated from different sources. Previous phylogenetic researches on *E. faecium* have identified two separate clades, representing the hospital-associated isolates (clade A) and community/commensal isolates (clade B) [12, 13]. A further split occurred within clade A, leading to the division of subclades A1 and A2 (corresponding to clinical and animal-associated isolates, respectively) [9]. Although the addition of the dairy and other accessible genomes of environmental isolates apparently changed the topology of the phylogenetic tree, the results of our phylogenetic analysis were principally in line with the observation of two separate clades (corresponding to the hospital- and community-associated clades), which are named here as clades I and II, respectively. Lebreton et al.’s study [9] further argued that a relatively recent split occurred within clade A to form clade A1 (containing mainly hospital-associated isolates) and clade A2 (containing mainly animal-associated isolates). Our analysis found that the animal-associated isolates were indeed dispersed in clades I and II, suggesting a greater genetic distance among these isolates than previously reported in Lebreton et al. Similar to the findings of Raven et al. [10] that analysed healthcare-associated *E. faecium* that covered a decade (2001–2011) of genomic history of the United Kingdom and Ireland, our results did not support the subdivision of clade A into clades A1 and A2 [9].

The fact that the dairy isolates were dispersed in all four subclades in the currently reconstructed phylogenetic tree though with uneven distribution suggests that they did not arise from a common recent ancestor. Some of the dairy isolates clustered together in the crown, particularly those allocated to subclades IB and IIA, existing as independent lineages. These results suggest that the dairy isolates existed as independent lineages rather than a product of faecal contamination, although the boundary is not entirely clear cut. Occasionally, isolates that were originated from different habitats clustered together. We speculate that some bacteria could be disseminated to habitats other than their original niches by chance and adapted to the new environment. For example, the dairy isolate XJ11301 clustered with human isolates in subclade IA. This might due to the access of dairy strains to the human intestinal tract possibly because of consumption of natural fermented milk. Conversely, the human isolate E1071 was distributed to subclade IB that contained mainly the dairy isolates; this could have been due to personnel contact or contamination in the process of fermented dairy food production. It also seems that some isolates can adapt to two or more habitats well, and they clustered in subclade IIB, consisting of both the dairy isolates and a hybrid branch of isolates of multiple origins.

Next, we noted that the bacterial isolates of the same source, e.g. dairy isolates, did not just cluster based on geographic proximity. The genomes of most of the dairy strains were sequenced by our laboratory (mainly collected from China and Russia), while other bacterial genomes were retrieved from a public database, including three Argentinean isolates (GM70, GM75, and IQ110), three Italian isolates (UC7267), one Norwegian isolate (E1604), one Algerian isolate (GL1), and one isolate of unknown geographic origin, respectively. Interestingly, the Argentinean isolates GM70 and IQ110 clustered with the Russian dairy isolate Ru39–5 and Xinjiang (China) dairy isolate XJ1306, while another Argentinean isolate GM75 clustered with the human isolate E1972. In addition, the Italian isolate UC7267 clustered with two Xinjiang dairy isolates XJ9302 and XJ9–4; the Norwegian isolate E1604 clustered together with the Inner Mongolian dairy isolate NM31–5; the Algerian isolate GL1 clustered with the dairy isolates of Tyva, Russia. These results demonstrated that habitat was a more determining factor than geographic proximity for phylogenetic relationship.

The current phylogenetic analysis included a putative ancient isolate, 58 M, which was originated from the GI of *M. primigenius* living in New Siberian Islands 28,610 ± 110 years ago [22]. Our data revealed that the 58 M isolate was located within subclade IB and clustered with some modern isolates of various origins. Such results suggest that the branch divergence occurred far earlier than the extinction of *M. primigenius*. Thus, our data do not support the conclusion of Lebreton et al. that the branch divergence represented the splitting of animal-originated isolates from the human commensal line approximately 3,000 years ago due to increasing urbanization of humans [9]. Instead, our results are more in line with the estimation of Galloway-Peña and colleagues that the divergence occurred about 300, 000 to 3, 000, 000 years ago [12]. By integrating the dairy isolates, the phylogenetic tree reconstructed here has broadened our knowledge and experience of the phylogenetic relationship among hospital-, community-, and animal-associated *E. faecium*.

Thirdly, results from our genome-wide association study identified 202 environment-specific genes, showing great genomic plasticity of *E. faecium*. One hundred and thirty-six of these genes were dairy-specific. Five of the dairy-specific genes were almost universally shared by isolates of dairy origin (> 95% of dairy isolates contained all five genes), which encoded five lactose metabolism associated genes (*lacA*/*B*, *lacC*, *lacD*, *lacF*, and *lacG*). For non-dairy isolates, the frequency of these genes decreased to 21%. It is noteworthy that these five genes constitute an integrated lactose metabolism pathway, which functions to transport the extracellular lactose into the cell and further catalyze it to D-glyceraldehyde 3-phosphate to be finally utilized in the glycolysis/gluconeogenesis pathway. Lactose is the major carbon source in milk; thus, it is sensible that almost all dairy isolates have retained these genes for optimization of energy acquisition and growth in milk. In contrast, isolates that were isolated from other ecological environments, such as GI and blood, mainly utilized non-lactose-based carbon sources and thus the lactose-utilization genes became dispensable. Although the phylogenetic analysis revealed that the host origin is a key determining factor in shaping the *E. faecium* genomes, the environment seems to play an important role too. Moreover, the large genome plasticity of *E. faecium* could contribute to the high versatility of this species in adapting successfully to a wide range of environments from dairy to harsh conditions such as the hospital environment and GI [23].

Finally, the increasing medical significance of the health-care associated *E. faecium* lineages has driven us to analyse the spectrum of antibiotic resistance genes in these genomes in detail. Cluster analysis based on the distribution of vancomycin resistance genes found three types of antibiotic resistance spectrum: *vanA*, *vanB*, and van absent (Fig. 5). The dairy isolates had the fewest antibiotic resistance genes and belonged to the van absent category, making them significantly different from the human and animal isolates. Such results also support that the dairy isolates were not derived merely from faecal contamination but existed as independent lineages. Antibiotic resistance has been acquired and disseminated throughout enterococci via horizontal transfer of mobile genetic elements [24]. The antibiotic resistance genes render human and animal isolates successful colonizers or even invaders of human and animal GI tracts [5], while the antibiotic selective pressure is much less in dairy products, resulting in a general absence of this spectrum of genes among the dairy isolates. Among the other two types of antibiotic resistance profile, the *vanA* type cluster is the most frequently encountered type of glycopeptide resistance in enterococci; and isolates that have acquired *vanA* are resistant to high levels of vancomycin [25]. The organization and functionality of the *vanB* cluster is similar to that of *vanA*, but the resistant levels are variable [25]. In this study, about three quarters of isolates from human and animals carried vancomycin resistance genes, which is more prevalent than *E. faecalis* [26].

## Conclusions

In conclusion, the comparative genomic study of *E. faecium* showed significant differences between isolates from dairy products and humans. The dairy isolates existed as independent lineages rather than a product of faecal contamination based on our phylogenetic analysis. Results from the phylogenetic and genome-wide association studies together revealed links between the bacterial isolation environment, physiological features, and phylogeny. Moreover, the great genomic plasticity of *E. faecium* probably provides the strong environmental adaptability to enable the successful adaptation of this species to various ecological niches.

## Methods

### Bacterial isolates and genomes

A total of 161 genomes of *E. faecium* were subjected to whole genome sequencing-based analysis. Among them, 54 isolates were previously isolated from naturally fermented dairy products in China and Russia from 2007 to 2014 by our laboratory; they were firstly sequenced in this study (Additional file 1). The other *E. faecium* genomes were retrieved from the Genbank database on April, 2017. At the start of this work, all accessible genome records of *E. faecium* were investigated to include only those having clear documentation of bacterial isolation source and of high sequencing quality. These two criteria were important to ensure comparability between genomes and grouping of isolation environment. After screening, 107 genomes remained, which were from human GI (33 genomes), human blood (39 genomes), human UT (9 genomes), chicken GI (8 genomes), dog GI (2 genomes), pig GI (7 genomes), *M*. *primigenius* GI (1 genome), dairy products (7 genomes), and soy (1 genome), respectively (Additional file 1).

The genomes of three *E. mundtii* isolates (accession numbers: AP013036, CP018061, and CP022340) were downloaded and included as outgroups in the phylogenetic tree reconstruction.

### DNA extraction

Bacteria were grown in de Man Rogosa and Sharpe (MRS) broth under anaerobic conditions at 37 °C. Bacterial DNA was extracted with a commercial DNA extraction kit (OMEGA D3350–02) according to the manufacturer’s instructions. The amount of extracted genomic DNA was quantified using a TBS-380 fluorometer (Turner BioSystems Inc., Sunnyvale, CA). Fragment libraries (200 to 300 bp) were constructed only with high-quality DNA (OD260/280 = 1.8~2.0, > 6 μg).

### Whole-genome sequencing, assembly, and annotation

The whole-genome sequencing was done using the Illumina MiSeq platform (Illumina Inc. U.S.A) by generating 2 × 150 bp paired-end libraries using the Nextera DNA Sample Preparation Kit (Illumina Inc., U.S.A) following the manufacturer’s instructions. On average, 1,576 Mb of high-quality data were generated for each isolate, corresponding to 395- to 969-fold sequencing depth (Additional file 1).

The paired-end reads were first assembled de novo using SOAPdenovo v1.06 [27]. GapCloser (http://sourceforge.net/projects/soapdenovo2/files/GapCloser/) was used to fill local inner gaps and correct single base errors. Glimmer v3.02 was used to predict putative coding sequences [28]. RAST 2.0 [29] and COG database [30] were used to annotate the functions of predicted open reading frames (ORFs). The assembled genomes of the 54 isolates were deposited in the National Center for Biotechnology Information GenBank database under the accession numbers of PGPI00000000 to PGTQ00000000 (Additional file 1).

### Construction of core- and pan-genomes

All predicted ORFs were firstly assigned to the respective gene families. Then, the core- and pan-genomes of *E. faecium* were constructed using the SiLiX software [31] on the basis of homologous gene families. Two ORFs would be assigned to the same family if the amino acid sequence identity was greater than 80% and if the shorter ORF aligned over 80% of that of the longer one. The pan-genome comprised the total number of non-redundant gene families within the complete dataset, while the core-genome included gene families which were present in all *E. faecium* genomes. The longest ORF from each gene family was used as the representative sequence for functional annotation and phylogenetic analysis.

### Construction of phylogenetic tree

The phylogenetic tree was constructed using the core genes of 161 *E. faecium* and three *E. mundtii* isolates. The species *E. mundtii* was the closest relative of *E. faecium* and was thus used as outgroups. Briefly, the nucleotide sequences of the core genes were aligned using MUSCLE v3.8.31 [32], and the unreliable alignment regions and intragenic homologous recombination were removed by using Gblocks (http://molevol.cmima.csic.es/castresana/Gblocks.html) and Gubbins (http://www.sanger.ac.uk/science/tools/gubbins) [33], respectively. A maximum likelihood tree based on the concatenated alignments was built using FastTree 2.1.8 [34] with 1,000 bootstrap iterations. The phylogenetic tree was visualized with the online tool, Interactive Tree Of Life (iTOL) [35].

### Identification of environment-specific genes

In order to identify environment-specific genes, the portion of variable genes found in the pan-genome was analysed to determine if there was significant association between gene distribution and bacterial isolation source. Environment-specific genes were defined as those having a significantly higher prevalence in isolates from one particular isolation source compared with the overall occurrence across all genomes. Scoary 1.6.16 was applied to identify environment-specific genes based on 1,000 permutation replicates. The results were corrected for multiple testing with Benjamini-Hochberg correction. A corrected *P*-value < 0.05 was considered significant [36].

### Identification of antibiotic resistance genes

A BLASTp search was conducted with the predicted genes from all investigated genomes against the CARD (Comprehensive Antibiotic Resistance Database; http://arpcard.mcmaster.ca) to identify potential antibiotic resistance genes (E-value <1e-15, coverage > 90% and sequence identity > 85%) [37, 38]. The hierarchical cluster analysis was based on the presence or absence of the antibiotic resistant genes and the heatmap was drawn using the “pheatmap” package of the R software (V3.6.0).

### Identification of virulence factors

A BLASTp search was done with the predicted genes from all investigated genomes against the VFDB (Virulence Factor Database) to identify genes related to potential virulence factors (E-value <1e-15, coverage > 90% and sequence identity > 85%) [38, 39].

### Statistical analysis

Data are presented as means ± SEM. One-way ANOVA followed by Tukey’s post-hoc test was used for statistical significance determination using the SPSS Statistics 19 (IBM, Armonk, New York, USA). Significance was set at *P* < 0.05.

## Additional files

Additional file 1: List of the *Enterococcus faecium* isolates analysed in this study. (XLSX 25 kb)

Additional file 2: The length distribution of coding DNA sequences (CDSs) in *Enterococcus faecium* genomes. (XLSX 62 kb)

Additional file 3: Clusters of Orthologous Groups (COGs) functional categories of predicted genes of dairy and human isolates. [J] Translation, ribosomal structure and biogenesis; [K] Transcription; [L] Replication, recombination and repair; [D] Cell cycle control, cell division, chromosome partitioning; [V] Defense mechanisms; [T] Signal transduction mechanisms; [M] Cell wall/membrane/envelope biogenesis; [N] Cell motility; [U] Intracellular trafficking, secretion, and vesicular transport; [O] Post-translational modification, protein turnover, and chaperones; [C] Energy production and conversion; [G] Carbohydrate transport and metabolism; [E] Amino acid transport and metabolism; [F] Nucleotide transport and metabolism; [H] Coenzyme transport and metabolism; [I] Lipid transport and metabolism; [P] Inorganic ion transport and metabolism; [Q] Secondary metabolites biosynthesis, transport, and catabolism; [R] General function prediction only; [S] Function unknown. (PDF 208 kb)

Additional file 4: Phylogenetic tree constructed based on the core genes of *Enterococcus faecium* isolates. Bootstrap values are shown at the nodes. (PDF 35 kb)

Additional file 5: The antibiotic resistance genes in the 161 isolates of *Enterococcus faecium (XLSX 71 kb)*

Additional file 6: The virulence factors in the 161 isolates of *Enterococcus faecium (XLSX 32 kb)*

Additional file 7: List of environment-specific genes (XLSX 30 kb)

## Data Availability

The individual genome assemblies of 54 isolates were deposited in the National Center for Biotechnology Information under accession numbers PGPI00000000 to PGTQ00000000 (Additional file 1).

## Abbreviations

*E*: *Enterococcus*
GI: Gastrointestinal
*M*.: *Mammuthus*
MRS: Man Rogosa and Sharpe
ORFs: Open reading frames
UT: Urinary tract
