# Supplementary material for: Comparative genomic analysis revealed great plasticity and environmental adaptation of the genomes of Enterococcus faecium
Source: BMC Genomics. 2019 Jul 22;20:602. doi: 10.1186/s12864-019-5975-8 (PMC6647102; doi:10.1186/s12864-019-5975-8)
Supplement: Supplementary file 3 — Clusters of Orthologous Groups (COGs) functional categories of predicted genes of dairy and human isolates. [J] Translation, ribosomal structure and biogenesis; [K] Transcription; [L] Replication, recombination and repair; [D] Cell cycle control, cell division, chromosome partitioning; [V] Defense mechanisms; [T] Signal transduction mechanisms; [M] Cell wall/membrane/envelope biogenesis; [N] Cell motility; [U] Intracellular trafficking, secretion, and vesicular transport; [O] Post-translational modification, protein turnover, and chaperones; [C] Energy production and conversion; [G] Carbohydrate transport and metabolism; [E] Amino acid transport and metabolism; [F] Nucleotide transport and metabolism; [H] Coenzyme transport and metabolism; [I] Lipid transport and metabolism; [P] Inorganic ion transport and metabolism; [Q] Secondary metabolites biosynthesis, transport, and catabolism; [R] General function prediction only; [S] Function unknown. (PDF 208 kb) [file 12864_2019_5975_MOESM3_ESM.pdf]

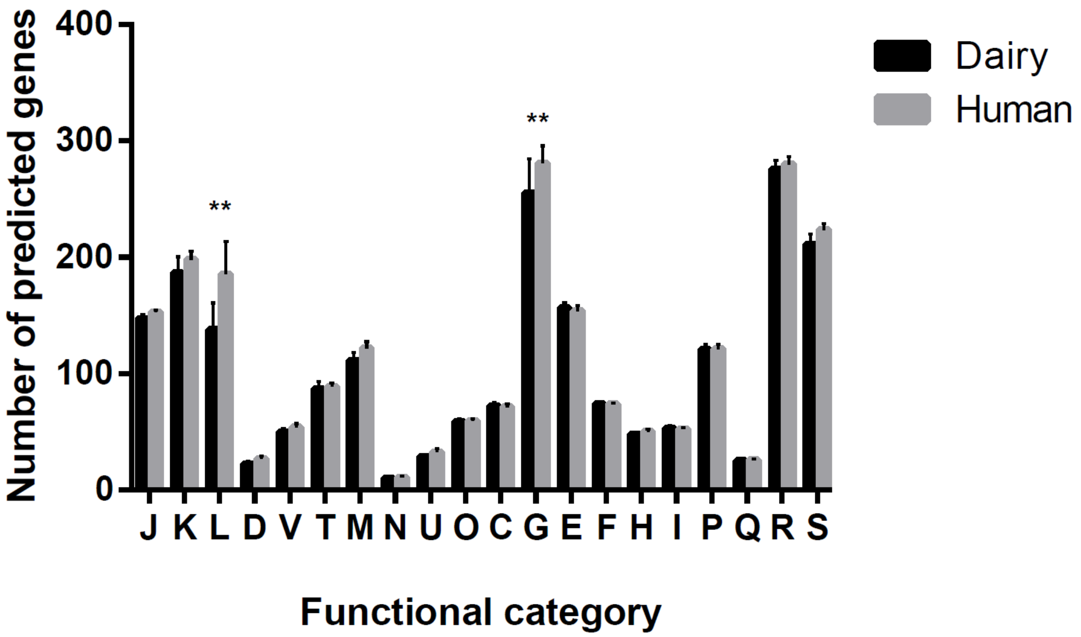

Additional file 3: Clusters of Orthologous Groups (COGs) functional categories of predicted genes of dairy and human strains. [J] Translation, ribosomal structure and biogenesis; [K] Transcription; [L] Replication, recombination and repair; [D] Cell cycle control, cell division, chromosome partitioning; [V] Defense mechanisms; [T] Signal transduction mechanisms; [M] Cell wall/membrane/envelope biogenesis; [N] Cell motility; [U] Intracellular trafficking, secretion, and vesicular transport; [O] Post-translational modification, protein turnover, and chaperones; [C] Energy production and conversion; [G] Carbohydrate transport and metabolism; [E] Amino acid transport and metabolism; [F] Nucleotide transport and metabolism; [H] Coenzyme transport and metabolism; [I] Lipid transport and metabolism; [P] Inorganic ion transport and metabolism; [Q] Secondary metabolites biosynthesis, transport, and catabolism; [R] General function prediction only; [S] Function unknown. “**” represents $P<0.05$.
