# Supplementary figures and images for: Comparative genomic analysis revealed great plasticity and environmental adaptation of the genomes of Enterococcus faecium
Source: BMC Genomics. 2019 Jul 22;20:602. doi: 10.1186/s12864-019-5975-8 (PMC6647102; doi:10.1186/s12864-019-5975-8)

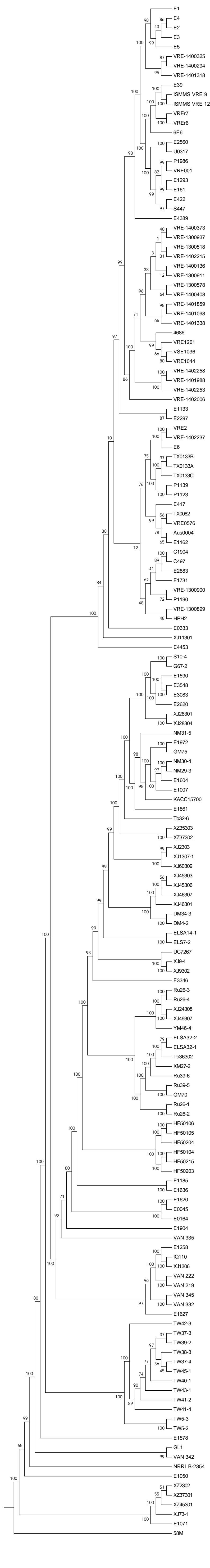

Supplement: Supplementary file 4 — Phylogenetic tree constructed based on the core genes of Enterococcus faecium isolates. Bootstrap values are shown at the nodes. (PDF 35 kb) [file 12864_2019_5975_MOESM4_ESM.pdf]
